# Ngāokeoke Aotearoa: The *Peripatoides* Onychophora of New Zealand

**DOI:** 10.3390/insects15040248

**Published:** 2024-04-04

**Authors:** Steven A. Trewick, Emily M. Koot, Mary Morgan-Richards

**Affiliations:** 1 Wildlife & Ecology, School of Natural Sciences, Massey University, Private Bag 11-222, Palmerston North 4410, New Zealand; m.morgan-richards@massey.ac.nz; 2 New Zealand Institute for Plant and Food Research Ltd., Palmerston North 4410, New Zealand; emily.koot@plantandfood.co.nz

**Keywords:** Onychophora, peripatus, *Peripatoides*, *Ooperipatellus*, velvet worm, allozyme, mitochondrial DNA, cryptic species

## Abstract

**Simple Summary:**

The phylum Onychophora has only about 200 described species around the world. Commonly known as velvet worms or peripatuses, they are soft-bodied, many-legged invertebrates. Onychophora hunt at night and live in moist places on land. On the outside, they all look very similar which makes species identification difficult. In Aotearoa, New Zealand, the species within the endemic genus of live-bearing *Peripatoides* are known as ngāokeoke. One species in this genus is distinguished by having 16 pairs of legs (*P. suteri*), while others have 15 pairs of legs. One species (*P. indigo*) has a distinctive blue colour, but other taxa have a mix of orange and blue pigmentation. Five northern species within *Peripatoides* were established from genetic evidence of reproductively isolated sympatric populations. Morphological variation in this genus is re-examined using additional sampling from North Island and South Island, New Zealand. A re-analysis of nuclear markers and new DNA sequences confirms that five species are cryptic and their known ranges have been updated. Three new ngāokeoke species in the genus *Peripatoides* are described from South Island. These three new species represent distinct genetic lineages with distinct pigmentation patterns.

**Abstract:**

(1) Background: Originally described as a single taxon, *Peripatoides novaezealandiae* (Hutton, 1876) are distributed across both main islands of New Zealand; the existence of multiple distinct lineages of live-bearing Onychophora across this spatial range has gradually emerged. Morphological conservatism obscured the true endemic diversity, and the inclusion of molecular tools has been instrumental in revealing these cryptic taxa. (2) Methods: Here, we review the diversity of the ovoviviparous Onychophora of New Zealand through a re-analysis of allozyme genotype data, mitochondrial DNA cytochrome oxidase subunit I sequences, geographic information and morphology. (3) Results: New analysis of the multilocus biallelic nuclear data using methods that do not require a priori assumptions of population assignment support at least six lineages of ovoviviparous *Peripatoides* in northern New Zealand, and mtDNA sequence variation is consistent with these divisions. Expansion of mitochondrial DNA sequence data, including representation of all existing taxa and additional populations extends our knowledge of the scale of sympatry among taxa and shows that three other lineages from southern South Island can be added to the *Peripatoides* list, and names are proposed here. In total, 10 species of *Peripatoides* can be recognised with current data.

## 1. Introduction

Living Onychophora, more commonly known as peripatuses or velvet worms, and in Aotearoa, New Zealand, by their te reo (Maori language) name ngāokeoke, are soft-bodied, segmented, predatory invertebrates [1,2,3]. The phylum is placed within the Panarthropoda, sister to the Arthropoda [4,5]. Onychophora lack a rigid exoskeleton instead, like annelids, relying on hydrostatic pressure inside a water-repellent cuticle to maintain body form. They are susceptible to dehydration through respiration water, and as a result, they are constrained to humid or moist environments and are nocturnal. They hunt a wide range of invertebrate prey in the open air when conditions are suitable or within decomposing logs, leaf litter and soil, in caves and under rocks [2,3,6].

Extant Onychophora comprise the equatorial Peripatidae and the southern Peripatopsidae in Central Chile, South Africa and Swaziland, Papua New Guinea, Indonesia, Australia and New Zealand [7,8]. The first onychophoran from Aotearoa, New Zealand, was described [2] fifty years after the first description of ‘a leg-bearing slug’ from Central America [1]. *Peripatoides novaezealandiae* (Hutton, 1876) was established without type material and characterised from specimens collected at three locations spanning the country: Wellington at the south of North Island, Nelson on the northwest of South Island and Dunedin in southeast South Island. The species was defined as having fifteen pairs of legs, three (sometimes four) spinous foot pads on the underside of each leg, and three (rarely four) distal papillae on the ends of its feet [2]. The length is variable from 2.5 cm to 5 cm, and the colour depends on the relative number of orange and blue–grey epidermal papillae. Hutton noted that the arrangement of these pigmented epidermal papillae was sometimes suggestive of lateral stripes or other patterns but varied within and between populations. He described the genital opening as circular, tumid, wrinkled, usually grey, or sometimes pale or white [2].

Since then, the taxonomy of ngāokeoke Aotearoa, New Zealand Onychophora, has relied primarily on colour and leg number. Thus, when reviewed by Watt in 1960 [9], the endemic Peripatopsidae comprised two live-bearing (ovoviviparous) *Peripatoides*, *P. novaezealandiae* (Hutton, 1876) with 15 pairs of legs, and *P. suteri* (Dendy, 1894) [10] with 16 pairs, and the egg-laying (oviparous) *Ooperipatellus viridimaculatus* (Dendy, 1900) [11] with 14 pairs of legs. A second egg-laying species, *O. nanus* (13 leg pairs), was added [12] along with a second live-bearing species with 15 pairs of legs (*P. indigo* Rhuberg, 1985), which is distinguished from *P. novaezealandiae* by its uniform colour, geographic distribution and number of distal foot papillae [12]. 

The live-bearing *Peripatoides* tend to be bigger than egg-laying *Ooperipatellus*, and this probably reflects heavy investment in relatively few young. Dissection of *P. novaezealandiae* individuals suggests that fertilisation involves dermal insemination whereby spermatophores placed on the female’s body release spermatozoa that pass through the cuticle to the haemolymph [13]. Inside the body, it seems they migrate to posterior spermathecae, where they can be found in mature females. Dermal or other transfer of spermatophores has not, however, been observed in *Peripatoides* but has been seen in some Australian and South American Onychophora (e.g., [14,15,16,17]).

**Figure 1:**
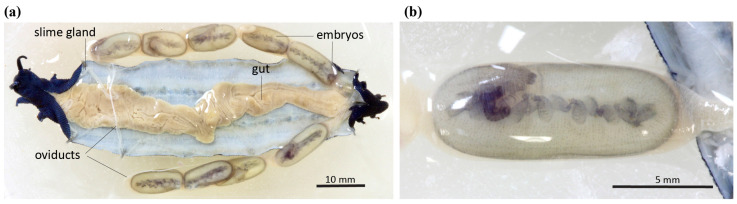
Internal anatomy of *Peripatoides indigo*: (**a**) dorsal view showing nine large embryos; (**b**) detail of a single late-stage embryo on which limbs are visible and pigment partly developed.

Females use the stored sperm to fertilise eggs within a pair of uterine tracts that loop from ovaries near the posterior, almost the length of the body and back to the posterior genital opening. Eggs seem to be fertilised in small groups as embryos are observed to be at a similar developmental stage (Figure 1). Juvenile *Peripatoides* possess little or no pigmentation when they emerge from their mothers, although females of the largest species, *P. indigo*, produce young at a more advanced, partially pigmented stage. The large size and the small number of developing embryos indicate high investment in few offspring, features shared with other K strategists [18], including in New Zealand, the kiwi *Apteryx australis* [19] with equilibrium populations in stable environments [20]. Female *P. novaezealandiae* collected near Wellington had broods averaging 12 embryos [13].

**Figure 2:**
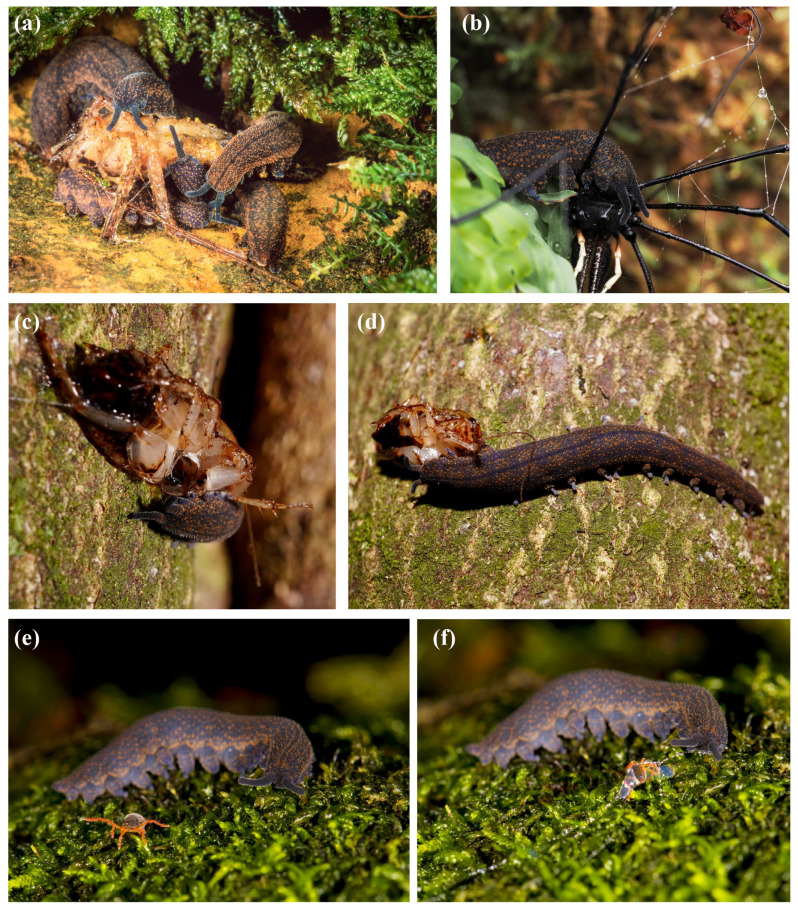
Nocturnal observations of *Peripatoides* feeding: Near the ground on, (**a**) Macropathinae cave wētā, Rod Morris; (**b**) Opilione *Pantopsalis*, Emily Roberts; (**c**,**d**) *Celatoblatta* cockroach on vertical tree trunk 2 metres above the ground; (**e**,**f**) Penthaleid mite, Thomas David Miles.

Nocturnal observations are revealing more about ngāokeoke activity, foraging and distribution (Figure 2). Unlike more arid environments where Onychophora may be more restricted to small, isolated habitats such as logs (e.g., [21]), the wetter conditions in most of New Zealand allow activity in the open. Remote monitoring in captivity indicates that *Peripatoides* maintain a circadian rhythm of about 24 h (Trewick unpublished) that is presumably entrained by external factors such as light and temperature. This implies that during the day, when concealed in suitable cool, damp recesses, they are probably inactive, and incidental observations suggest a compact resting state often compressed with legs drawn together is typical. Daytime roosting is not restricted to the ground as ngāokeoke have been detected in artificial wētā roosts (e.g., [22]) and natural tree holes (pers. obs).

If ambient conditions in the external environment are suitably cool and damp, ngāokeoke appear to be very active on the ground, among leaflitter and on rocks and trees. In all probability, these animals roam throughout the three-dimensional domain of the forest, especially where epiphytic plants and lichen are present. At night, it can be presumed that foraging activity below ground and in decaying logs involves searches for suitable prey in recesses of these equitable conditions. As observed in captivity, these predators feed on arthropods of all types using the ejection of a proteinaceous slime to entangle and subdue prey (Figure 2). Sharp jaws incise the prey, and digestive saliva is injected into the body cavity [23]. Feeding may take several hours as partial external digestion is followed by ingestion, although this activity is rarely observed in nature.

Analysis of *Peripatoides* with multilocus nuclear markers resulted in the proposal to subdivide *P. novaezealandiae* sensu (Hutton, 1876) into a number of taxa [24,25], and subsequent analyses of mitochondrial DNA sequence data provided additional evidence for the lineages proposed along with additions from southern South Island [26,27]. The genotypic data resolved a signal for five distinct lineages within the putative *P. novaezealandiae* morphotype consistent with their species status: *P. novaezealandiae*, *P. aurorbis*, *P. kawekaensis*, *P. sympatrica,* and *P. morgani*. Critically, allelic variation at 17 independent, expressed nuclear loci revealed locations in North Island where distinct genotypes existed in close sympatry. This finding demonstrated that a strict biological species concept could be applied as the data show a lack of gene flow between co-occurring populations of ngāokeoke having the opportunity to interbreed if capable. This resulted in a complex hypothesis of overlapping and sometimes disjunct spatial distribution that is not readily resolved using macro morphological characters. Colour pattern varies considerably across much of this range but does not, on its own, reliably resolve species limits. 

The correlation of signal from single locus mitochondrial sequence data and functional, multilocus, biparental genotype data supports the use of the former when resolving *Peripatoides* lineages and identifying range overlap among these morphologically cryptic taxa. Here, we re-analyse those nuclear data, expand short DNA sequence data representing additional sample locations, and examine external morphological variation within and among putative taxa. We clarify the current knowledge of *Peripatoides* diversity in New Zealand and their sympatry and propose names for three additional southern lineages. 

## 2. Materials and Methods

Ngāokeoke were collected by hand throughout New Zealand, euthanised via freezing and stored in 95% ETOH. The habitats from which these specimens were collected during the daytime included native and exotic decomposing logs, amongst humus and soil beneath native vegetation and under rocks, and from cave walls and trees at night. In total, 202 individuals were used in this study, including those previously reported [26,27]. Sampling locations were mapped in R version 4.2.2 [28] using the *maps* and *mapdata* packages [29,30].

Allozymes are allelic variants of enzyme proteins comprising sequences of amino acids. The net electrical charge of variants allows them to be distinguished using electrophoresis and be revealed by staining that utilises the biochemical pathway of the protein concerned [31]. Allelic variants result from non-synonymous nucleotide substitution in contrast to the primarily synonymous nucleotide variation found in most DNA sequence markers used in population studies. As previously reported, allele frequency data were obtained from 17 putative allozyme loci in 109 *Peripatoides* individuals collected primarily in North Island, New Zealand [26,27]. We analysed these data using an ancestral admixture approach implemented in the R package LEA [32]. We used the snmf function with the parameter settings: K = 1:10, entropy = T, ploidy = 2, repetitions = 10, tolerance = 0.00001. This is a statistically naive approach that estimates individual admixture coefficients from the genotypic matrix after determining the optimal number of ancestral populations (clusters). The simultaneous optimisation of cluster number and assignment allows the identification of groups of individual genotypes independent of other priors. Resulting sNMF (sparse non-negative matrix factorisation) plots show estimated individual ancestry to genotypic clusters based on the signal from input data. 

One or two legs dissected from specimens were subject to DNA extraction using the solvent-free Proteinase K and salting-out method [33] or the Quantbio (Beverly, MA, USA) Extracta reagent. Partial 3′ cytochrome oxidase subunit I (COI) mitochondrial DNA sequences were generated using polymerase chain reaction with the primers C1-J-2195 [34] and NotLEUr [26], standard reagents and thermal cycling conditions [26,27]. Sanger sequencing was processed on a capillary ABI3730 Genetic Analyzer (Applied Biosystems, Foster City, USA). Sequences were edited using Geneious Prime [35], and resulting alignments of published and new data were used for phylogenetic analysis with Geneious and IQTree2 v2.2.2.6 [36] and minimum spanning network [37] implemented in PopArt v1.0 [38]. Diversity statistics were calculated in DnaSP v6.0 [39].

**Figure 3:**
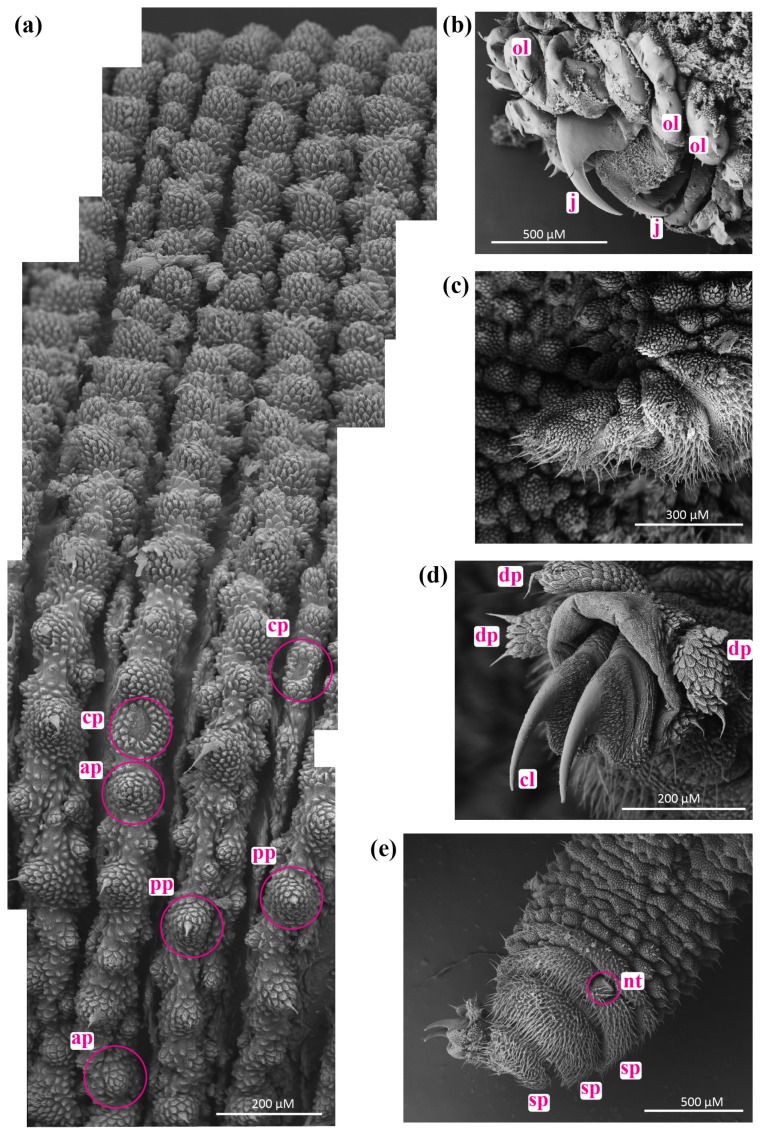
External micromorphology of ngāokeoke Aotearoa, New Zealand *Peripatoides*: (**a**) Swathe of abdominal papillae from left flank to dorsal midline with examples of primary papilla (pp), accessory papillae (ap) and crater papillae (cp); (**b**) jaws (j) surrounded by oral lips (ol) of preoral aperture or mouth; (**c**) lateral view of left oral or slime papilla; (**d**) foot showing distal papillae (dp) and claws (cl); (**e**) ventral view of 4th leg showing spinous pads (sp) and nephridial tubercle (nt).

**Figure 4:**
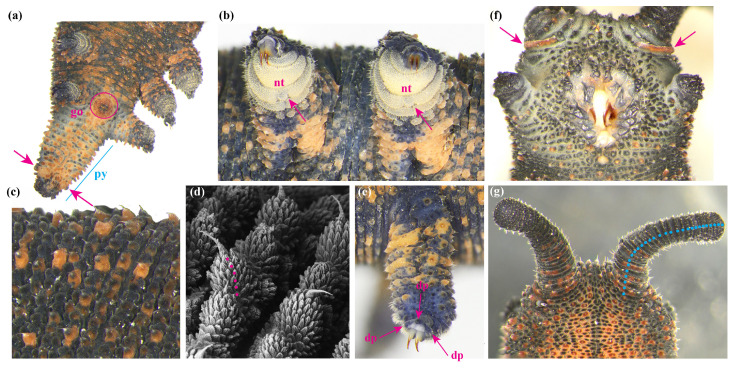
Morphological traits of *Peripatoides*: (**a**) Genital opening (go) and enlarged papillae (arrows) on pygidium (py) of a male; (**b**) nephridial tubercles (nt) in 3rd spinous of 4th and 5th legs; (**c**) region of cuticle with mixture of predominantly orange and predominantly blue–grey papillae and some of mixed pigment; (**d**) scale number on dermal papillae (pink spots); (**e**) leg showing distal papillae (dp); (**f**) ventral view of head showing orange antennal ring (arrows); (**g**) antennal rings (blue spots).

Specimens of northern *Peripatoides*, including representatives of *P. suteri*, *P. kawekaensis*, *P. morgani*, *P. novaezealandiae*, *P. aurorbis, P. indigo* and *P. sympatrica*, were examined to determine whether fixed morphological differences existed. Each specimen was taxonomically designated by its phylogenetic placement. Characters that have been reported as variable in these and other Onychophora (e.g., [12,40,41,42,43]) were examined in individuals: the number of leg pairs; the colour ratio of predominantly blue–grey or orange papillae; the extent of an orange antennal base; pigmentation of the genital opening; the number of distal papillae on the legs; the number of spinous pads on the legs; the full or partial division of a foot pad by a nephridial tubercle on the 4th and 5th pair of legs; the number of antennal rings counted from the first complete ring by the eye; the average number of ‘scales’ on dorsal papillae behind the final pair of legs; body length; and the presence/absence of a regular pattern of differently coloured papillae on the ventral, dorsal and lateral surfaces of each individual (Figure 3). Males were identified by the presence of a pair of glands, one on either side, towards the tip of a relatively long pygidium. The proportion of papillae of different colours was assessed by eye for a patch of 100 adjacent papillae on one latero-dorsal surface from between the bases of the 4th and 5th leg pairs up to the dorsal midline. As pigmentation is not precisely partitioned by structure, the colour of each papilla reflected the dominant colour from a perpendicular view. Scale numbers on papillae were counted from base to apex on each of the 10 papillae on the dorsal pygidium (Figure 4). We note that storage in ethanol has little impact on the pigmentation of these Onychophora.

## 3. Results

### 3.1. Allozyme Diversity

Among the sample of 106 North Island *Peripatoides*, we found statistical support for at least six or seven clusters. These clusters were characterised by fixed allele frequency differences among sets of individuals. Within the sample, individuals with 16 pairs of legs formed a distinct genetic cluster consistent with the species *P. suteri* (Dendy 1894). A similar level of genetic difference was detected among other genetic clusters comprising individuals with 15 pairs of legs and consistent with the species previously proposed [25]. 

**Figure 5:**
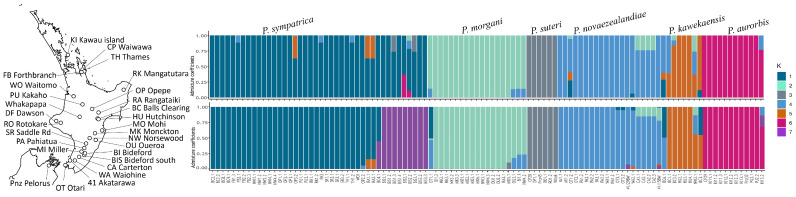
LEA sparse non-negative matrix factorisation of allozyme data showing individual North Island *Peripatoides* ancestry assignment. Coloured stacked bars indicate individual coefficients for each of six (above) or seven (below) hypothetical clusters.

This naïve Bayesian analytical approach has the advantage over traditional population genetics of not requiring a priori hypotheses about putative clusters. For *Peripatoides*, the results translate to patterns of shared ancestry such that some location samples comprise individuals of differing genotypes that each assigns to distinct clusters along with individuals from other location samples. For example, some individuals found at Balls Clearing belong to the *P. kawekaenis* cluster, while others from the same location belong to the *P. sympatrica* cluster (Figure 5). 

**Figure 6:**
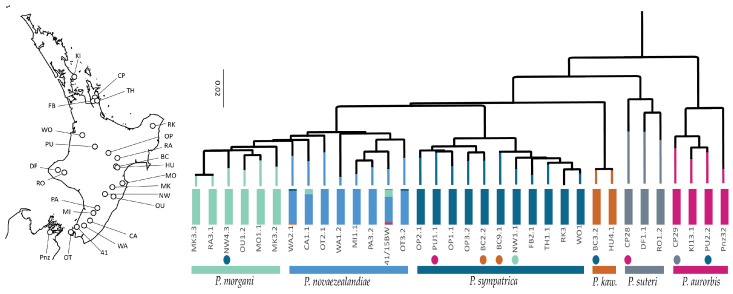
Multilocus nuclear genotypes (coloured vertical bars) and mtDNA variation (phylogeny) among a set of North Island *Peripatoides*. Individuals collected in sympatry are indicated by spots coloured for the corresponding cluster. Abbreviation *P. kaw*. refers to *P. kawekaensis*. Location abbreviations are as given in Figure 5.

For the samples with both types of data, allozyme and mitochondrial clusters correspond. However, as previously noted, under this scheme, *P. novaezealandiae* is paraphyletic with respect to *P. morgani* in terms of the mitochondrial clustering that might result from incomplete lineage sorting (Figure 6). 

### 3.2. Phenotypic Variation

**Figure 7:**
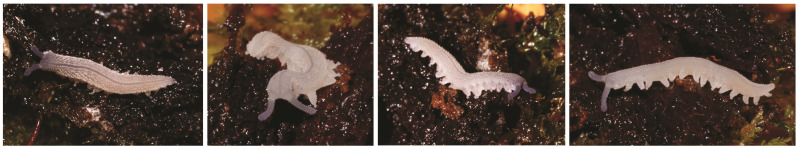
*Peripatoides* usually emerge from their mothers without pigmentation, which gradually develops as the young ngāokeoke grow. These *P. indigo* individuals have started to develop pigmentation on the antennae and dorsal midline.

**Figure 8:**
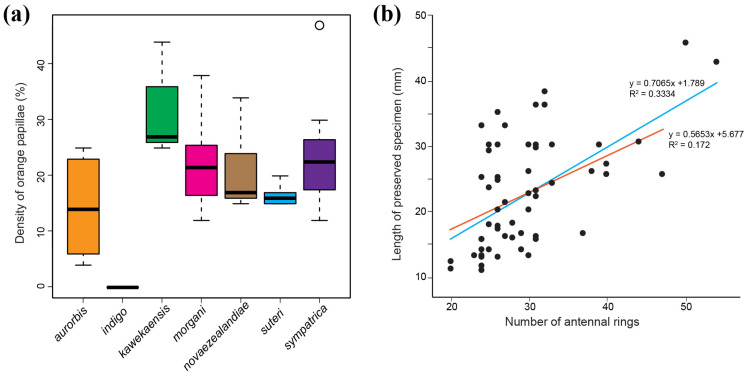
Morphological variation among *Peripatoides* species from central and northern New Zealand (see Table 1): (**a**) Variance in density of orange (versus blue–grey) papillae on the dorsal surface; (**b**) relationship between size of preserved specimens and their number of antennal rings among all individuals (blue) and all except two largest *P. indigo* (red).

Fifty-seven northern *Peripatoides* representing seven lineages classified by genetic data were examined for morphological differences and species/clade-defining characters. As colour develops gradually after the emergence of young from their mother (Figure 7), only adults with fully developed pigmentation were considered. Among these, all had 15 pairs of legs except *P. suteri*. All *P. suteri* had four distal papillae on most or all of their legs, and *P. indigo* had five (or more) (Table 1). Other species generally had three distal papillae, although sometimes four, and in all cases, there was some variation in the number and shape of the distal papillae among legs on an individual. All taxa had three entire spinous foot pads, often with an additional fragmented pad, apart from *P. indigo*, which had at least four entire pads. Other than *P. indigo*, all individuals of the other northern *Peripatoides* had some orange dermal papillae, with the density above the legs ranging from 5% to 47% (Figure 8, Table 1). 

Orange ocular papillae were absent from *P. indigo* and some *P. aurorbis* but present in others. Similarly, none of the *P. indigo* and *P. aurorbis* examined had orange colouration around the bases of their antennae, whereas individuals of other taxa displayed partial or complete orange antennal bases. All *P. aurorbis* examined in this subsample bore the orange genital opening described [25], although this was sometimes very pale. Some individual *P. suteri*, *P. sympatrica*, *P. morgani*, and *P. kawekaensis* had some orange pigmentation around the genital opening, though none displayed the intense contrast with a predominantly blue body colour of *P. aurorbis* examined from Kawau Island (the type locality) and the central North Island (Table 1). Most but not all individuals in all taxa had a visible nephridial tubercule on the 4th and 5th legs on both sides (Figure 3 and Figure 4). Where visible, this structure partially or completely bisected a spinous pad (Table 1).

Partial or complete division of a spinous pad on the 4th and 5th pairs of legs by a nephridial tubercle was apparent in all of the taxa. Similarly, the number of antennal rings, papillae ridge number, and lateral, ventral, and dorsal colour patterns showed overlapping variation among taxa (except the absence of orange in *P. indigo*) (Figure 8a). Therefore, the traits examined were revealed as unable to provide definitive diagnostic morphological characters for the genetic lineages investigated. However, this does not preclude the possibility that microscopic features are present. Variation in the length of specimens brought about by preservation reduces the value of size comparison with other traits, such as antennal ring number, and the hydrolastic nature of these animals causes individual appearance to vary considerably depending on conditions (Figure 8b).

**Figure 9:**
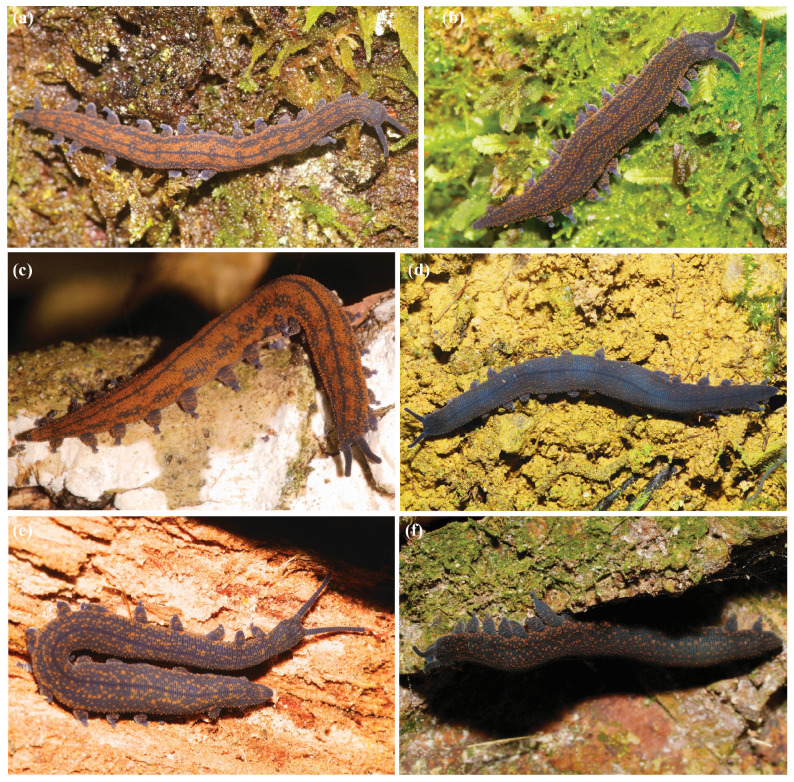
Colour pattern in *Peripatoides*. These adult *P. novaezeandiae* were photographed at a single location near Wellington and illustrate within-population variation reflecting different ratios and arrangements of blue–grey and orange papillae. (**a**,**c**,**e**) Strong, (**b**) partial, and (**d**,**f**) no pattern (assessed subjectively—See Table 1). Photographs courtesy of Uwe Schneehagen.

Examination of individuals belonging to a single lineage and from a single location illustrates the tendency for the northern *Peripatoides* to vary in colour pattern (Figure 9). These patterns appear to result from minor changes in the number and occurrence of two main colours (blue–grey and orange), forming a repeat pattern that coincides with leg pairs/segments. Areas lacking in pigmentation occur on the underside of the abdomen and under the legs (lobopods). Although these animals are nocturnal and the specific colour pattern unlikely to be of great ecological consequence, daytime foraging predators are likely to disturb resting ngāokeoke. Dark and orange colours may favour crypsis amongst forest leaf litter, soil and decaying wood.

**Figure 10:**
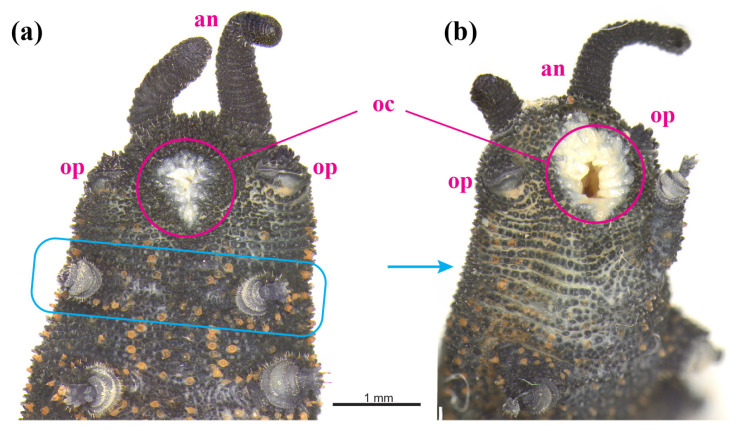
Anterior ventral surface of two *Peripatoides suteri* individuals from the same location and with the same genetic signature, showing oral papillae (op), antennae (an), oral cavity (oc). (**a**) First leg pair highlighted by blue box of a normal individual with 32 legs, and (**b**) an individual missing one of the first pair (blue arrow).

As well as colour variation, it is possible that leg number is more labile than assumed. One individual confirmed genetically to be *P. suteri* that came from the expected core range of the species in Taranaki had the expected 16 legs on the right side of the body, but only 15 on the left (Figure 10). This might be due to mechanical damage, but current knowledge indicates limited capacity for cuticular repair in Onychophora [44]. 

### 3.3. Phylogenetic Diversity

**Figure 11:**
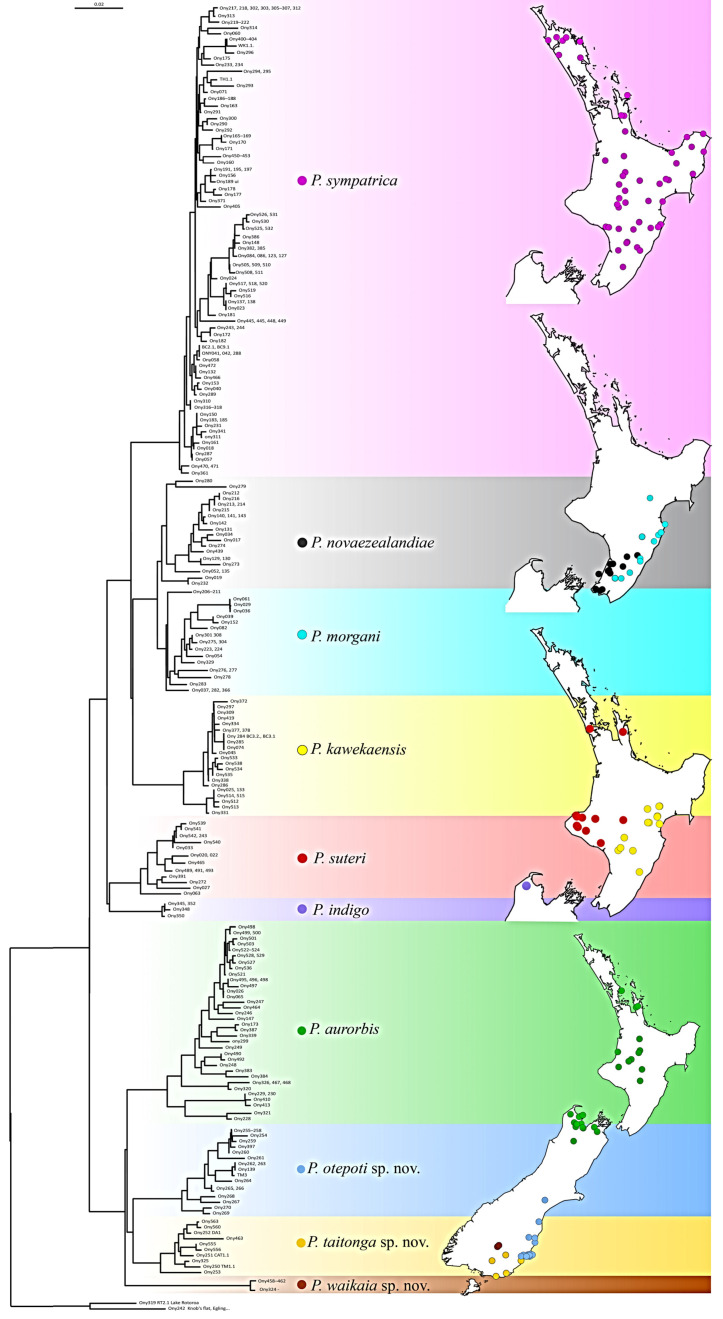
Neighbour-Joining phylogeny showing clusters of mitochondrial DNA COI haplotypes of New Zealand *Peripatoides*. Accompanying maps show approximate locations of sampling used in the analysis.

Analysis of a 525 bp alignment of sequences (GenBank: AF188241–188248, 188251–188254, 188258–188262, 221447–221497, PP135064–PP135297) representing 309 (Table S1) ingroup individuals helps resolve lineage assignment and geographic ranges of taxa (Figure 11). 

**Figure 12:**
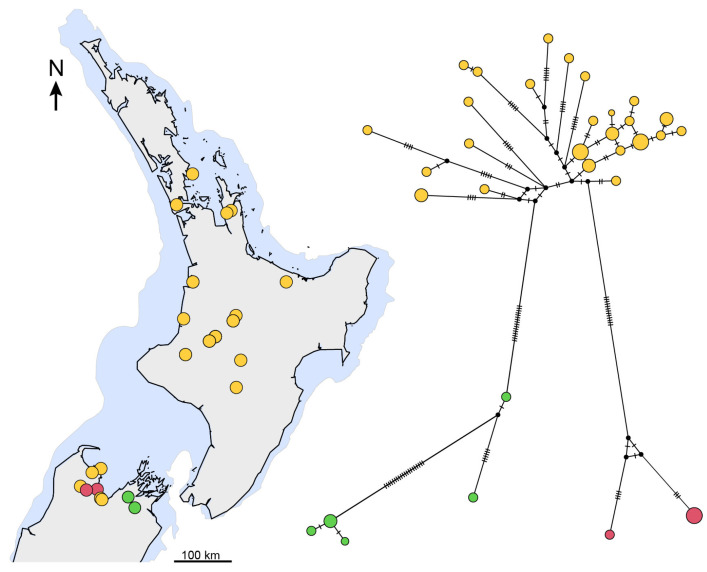
*Peripatoides aurorbis* sampling locations and median-joining network of 525 bp mtDNA COI sequences from 43 individuals. Haplotype cluster detected at each sample location is shown by colours. The estimated land surface during Pleistocene last glacial maximum (~28 ka) is indicated in blue [45].

With the exception of *P. waikaia* sp. nov., which was sampled from only one location, the lineages show high haplotype diversity, suggestive of large populations (Table 2). Nucleotide diversity, which gives an indication of the temporal depth of lineage population size, was highest in *P. aurorbis* (0.0333) and lowest in the two most narrowly sampled species, *P. indigo* (0.00191) and *P. waikaia* sp. nov. (0.00127). We note that *P. sympatrica*, which has a wide distribution across North Island (Figure 11), does not have especially high genetic diversity (Table 2) and this could be explained by the relatively recent range expansion from a smaller ancestral population. In contrast, additional sampling and mtDNA data show that the *P. aurorbis* lineage is present through North Island and on both sides of Cook Strait. DNA sequence diversity in this taxon reached 6.5% divergence (average 3.3%) across this range, but it is notable that considerably higher nucleotide diversity (Pi = 0.489) exists in South Island compared to North Island samples (Pi = 0.0142). This disparity in nucleotide diversity is explained by the full spectrum of *P. aurorbis* haplotype diversity being present in the northern South Island but only a subset of closely related haplotypes being present across North Island. Recent northward range expansion could result in this outcome (Figure 12).

**Figure 13:**
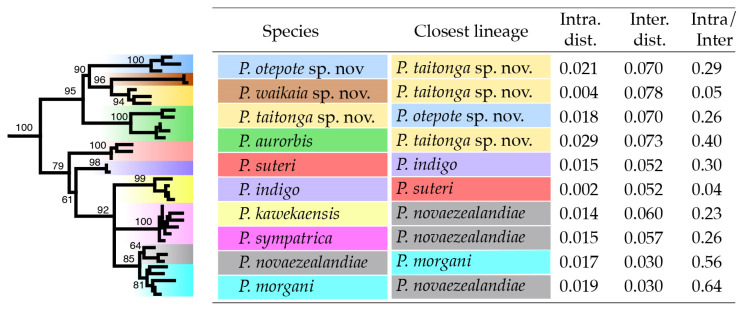
Genetic distances among ten *Peripatoides* lineages from mtDNA COI sequences derived with the Species Delimitation tool [46] in Geneious. Intraspecific distance refers to the focal species, and interspecific distance is the average between the focal and the closest lineage. Guide tree from IQTree with codon partition and 1000 bootstrap replicates.

Allied to *P. aurobis* (Figure 11 and Figure 13) are the three southern lineages, and this relationship is consistent with a northward range expansion of *P. aurorbis*. Nevertheless, the spatial separation of *Peripatoides* to either end of South Island suggests extinction or rarity in the central South Island. The most closely related taxa based on mtDNA COI sequences are *P. novaezealandiae* and *P. morgani* in North Island (Figure 13), with the lowest mean pairwise interspecies distances of 3%. Other comparisons exceed this considerably and reach 7.8% between two of the southern taxa.

### 3.4. Existing Species

*Peripatoides novaezealandiae* (Hutton, 1876). The species was defined as having fifteen pairs of legs, three spinous foot pads on the underside of each leg, and three distal papillae on the ends of its feet. Length is variable from 2.5 to 5 cm, and colour depends on the relative number of orange and blue–grey epidermal papillae. Hutton (1876) [2] described the genital opening as circular, tumid, wrinkled, usually grey, or sometimes pale or white. He also noted that the arrangement of these pigmented epidermal papillae was sometimes suggestive of lateral stripes or other patterns but varied within and between populations, although this, in part, resulted from him considering individuals from Wellington, Nelson and Dunedin that actually represented different species. No holotype or other material was recorded. Genetic evidence shows that a single species found in the Wellington region, *Peripatoides novaezealandiae*, is restricted to southern parts of North Island, New Zealand. This species shows particularly high colour polymorphism within locations (Figure 9).

Type material: Neotype Te Papa Tongarewa AI.012621. Voucher Te Papa Tongarewa AI.071927 (MPN-ONY135). Wilton’s Bush, Otari, Wellington, New Zealand (−41.2655, 174.755833). S.A.Trewick 2007.

*Peripatoides* suteri (Dendy, 1894). Ngāokeoke with 16 pairs of legs with four (or three) distal foot papillae and three spinous pads. Dendy (1894) proposed this taxon as a variety of *P. novazealandiae* restricted to Mt Taranaki [10] but did not designate type material. Subsequent analyses [24,25] confirm this is a distinct lineage (judged by leg number, number of distal papillae, allozymes and mtDNA sequences). This species was collected elsewhere on North Island, in the Central Plateau, Waitakere ranges, and the Coromandel Peninsula. Additional eastward occurrence is documented here. No holotype or other material was recorded, but syntypes exist in Hamburg (ZMH) and Paris (MNHN) [12].

Type material: Voucher Te Papa Tongarewa AI.071929 (MPN-ONY543). Dawson’s Falls, Mount Taranaki, New Zealand (−39.325089, 174.105781). M. Morgan-Richards 2021.

**Figure 14:**
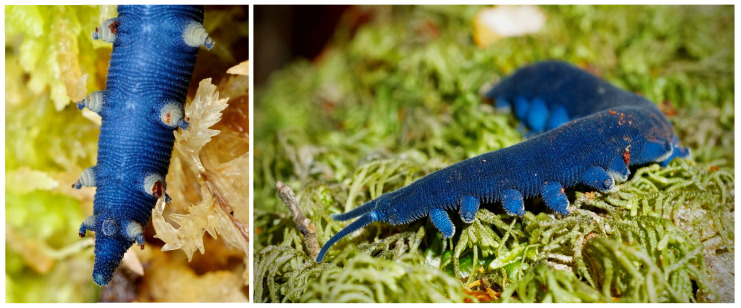
Female *Peripatoides indigo* from near Brown Hill, South Island, New Zealand.

*Peripatoides indigo* Rhuberg, 1985. Ngāokeoke with 15 pairs of legs, having five distal foot papillae, at least four spinous pads, and uniform indigo/blue colour [12] (Figure 14). It is restricted to the northwest corner of South Island known from the type locality near Paturau and near Brown Hill (newly reported here), which suggests the species range at least spans the Wakamarama Range. 

Type material: Holotype NZ Arthropod Collection NZAC03015391, Twin Forks Cave, Paturau. Voucher Te Papa Tongarewa AI.071928 (MPN-Ony351), near Brown Hill, Kahurangi, New Zealand (−40.90176, 172.43303), S.A. Trewick.

**Figure 15:**
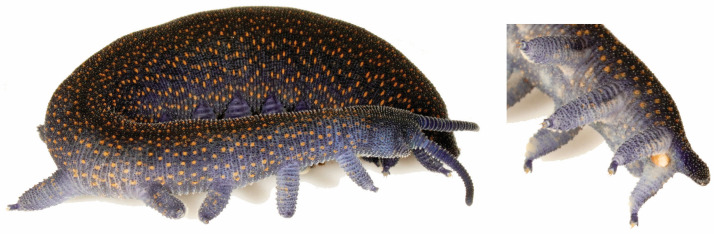
Female *Peripatoides aurorbis* with typical dominant blue–grey colour with evenly scattered orange papillae, pale underside and prominent orange genital opening.

*Peripatoides aurorbis* Trewick, 1998. Ngāokeoke with 15 pairs of legs bearing four distal papillae and three spinous pads, and typically with a bright orange genital opening that contrasts with a predominantly blue–grey background and evenly scattered orange papillae. This pattern is present throughout the species range but is most striking in northern populations (Figure 15), and where a higher density of orange is present, these are usually on the flank above the base of the legs. Next to *P. indigo*, *P. aurorbis* is the bluest of the *Peripatoides*. It has a spatial range that spans the Cook Strait and has the highest genetic diversity in the northern South Island. In North Island, its range partially overlaps with *P. sympatrica* and *P. suteri*.

Type material: Holotype Te Papa Tongarewa AI.012621. Kawau Island, New Zealand (36 30.00 S, 174 40.00 E). Voucher Te Papa Tongarewa AI.071923 (MPN-Ony523). Te Maire Valley Road, Whanganui, New Zealand (−38.951146, 175.188235), M. Morgan-Richards 2021.

*Peripatoides kawekaensis* Trewick, 1998. Ngāokeoke with 15 pairs of legs bearing three distal papillae and three spinous pads. Characterised by a unique fixed allele at the Aat-c locus [25]. It is found in the eastern North Island, including parts of Hawkes Bay and Manawatu. It is known to co-occur with *P. morgani* and *P. sympatrica*. 

Type material: Holotype Te Papa Tongarewa AI.012622. Hutchinson Reserve, Hawkes Bay (−39.266667, 176.533333), S.A. Trewick. Voucher Te Papa Tongarewa AI.071925 (MPN-Ony338), Maungatauwha, Hawkes Bay (−38.86247, 176.88714), M. Lusk 2013.

*Peripatoides morgani* Trewick, 1998. Ngāokeoke with 15 pairs of legs bearing three distal papillae and three spinous pads characterised by allozyme data. A majority of populations have been found to be fixed for different alleles at the Pk and Aat-a loci compared with other 15-legged ngāokeoke [25]. *Peripatoides morgani* occurs in southeast North Island (Figure 11) and is sympatric with *P. sympatrica* and *P. kawekaensis* Hawkes Bay and meets *P. novaezealandiae* in the Wairarapa. 

Type material: Holotype AI. 012623. Mohi Bush, Hawkes Bay, New Zealand. M. Morgan-Richards. Voucher Te Papa Tongarewa AI.071926 (MPN-Ony304) Mohi Bush, Hawkes Bay, New Zealand (−39.857376, 176.903057), M. Morgan-Richards 2012. 

**Figure 16:**
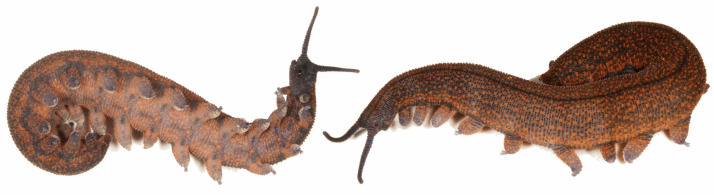
Typical *P. sympatrica* with a high density of orange papillae on all parts of the body amid relatively dark grey–blue. A dark dorsal midline is often apparent.

*Peripatoides sympatrica* Trewick, 1998. Ngāokeoke with 15 pairs of legs bearing three distal papillae and three spinous pads characterised by allozyme data and so named because of its wide distribution throughout North Island, which results in sympatry with other species ranges (Figure 16). *Peripatoides sympatrica* has a near-exclusive Acon A allele, with *P. novaezealandiae* and *P. kawekaensis* also having this allele, however only at a single location each and at very low frequencies [25]. It is known to be sympatric with *P. kawekaensis* and *P. morgani* in Hawkes Bay and with *P. aurorbis* in the central, west, and northern North Island.

Type material: Holotype Te Papa Tongarewa AI.012625 ANZAC Reserve, Norsewood, Hawkes Bay (−40.056264, 176.222677). S. A. Trewick. Voucher Te Papa Tongarewa AI.071924 (MPN-Ony153) ANZAC Reserve, Norsewood, Hawkes Bay. S. A. Trewick 2005.

### 3.5. New Species from the South

Hutton (1867) described a single taxon of *Peripatoides* based on specimens from Dunedin, Nelson and Wellington and identified that colour differences as reflecting variation in numbers of orange and blue/grey papillae. Ngāokeoke found today in Dunedin have a high proportion of orange papillae and show less variation within a population than seen in *P. novaezealandiae* around Wellington. 

Two taxa with their spatial ranges near Otepote—Dunedin are informally recognised [26,47,48], along with a third known to occur further inland in the region. These are here given formal names with their distinction based on geographic, phylogenetic and colour pattern information.

Three distinct mtDNA clusters have been apparent in the southern part of South Island of New Zealand for some time [24,26]. Current sampling that includes some additional locations indicates that two are parapatric with their juncture a little south of Dunedin City [26]. The third is known only from an inland forest remnant around the headwaters of the Waikaia River (towards the end of Piano Flat Road). 

With reference to the combination of nuclear and mitochondrial data obtained from northern *Peripatoides*, there is sound reasoning to infer at least three distinct species exist among the southern *Peripatoides*. As these entities are fairly widely recognised, it is appropriate to formalise names. 

***Peripatoides taitonga* sp. nov.**

Type locality: Croydon Bush, Southland, New Zealand.

**Figure 17:**
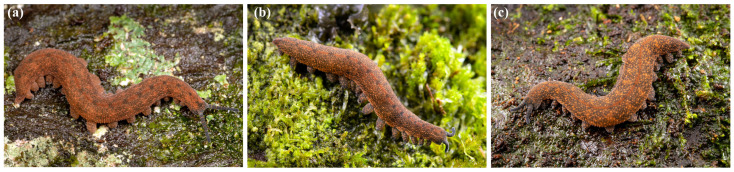
*Peripatoides taitonga* sp. nov. individuals from three locations across the species range: (**a**) Croydon Bush; (**b**) Seaward Downs; (**c**) Waipori. © Rod Morris.

Description: Ovoviviparous ngāokeoke with 15 pairs of legs bearing three distal papillae and three spinous pads. Third spinous pad on 4th and 5th legs completely divided by nephridial tubercle. Adult resting length up to about 30 mm. Colour dark grey/orange–brown (similar to Fe_2_O_3_ iron oxide) comprising a mixture of pigmented papillae that generally do not form a strong pattern (Figure 17). Some individuals display a predominantly orange dorsal midline with subtle to bold dark spots. Antennae primarily dark grey.

Most closely related to *P. aurorbis*, *P. otepoti* sp. nov., *P. waikaia* sp. nov. but differs from nearest (*P. otepoti* sp. nov.) by 7.3% at the mitochondrial COI gene. Intraspecific diversity in the present data set is 2.1%.

Material examined: MPN-Ony560–563, Black Gully, Tapanui (−45.89388, 169.35048); MPN-Ony551–554, Croydon Bush (−46.0633, 168.87371); MPN-Ony251, Haldane (−46.60722, 169.01556); MPN-Ony252, Hokonui (−46.057617, 168.825781); MPN-Ony325, Matai Falls (−46.49821, 169.49747); MPN-Ony555–559, Seaward Downs (−46.36253, 168.70472); MPN-Ony250, MPN-Ony576, Taieri Mouth (−46.05256, 170.19057); MPN-Ony253, Tom’s Creek (−45.851234, 169.471498); MPN-Ony463, Waipori Gorge (−45.915392, 169.992743). 

Distribution: Southern South Island, in the Southland district and southern part of Otago. This species meets *P. otepoti* sp. nov in Otago in the vicinity of the Taieri River and occurs on the northern bank of the Waipori River near Waipori Falls. The known range extends from the Catlins coast in Otago inland at least as far as the Blue Mountains and the eastern Hokonui Hills and south to Seaward Downs east of Invercargill, Southland. 

Etymology: Te reo (indigenous Maori language) taitonga means south or southern, a reference to the geographic distribution of this species. The name also alludes to Noel Tait, who was instrumental in revealing diversity within *Peripatoides*.

Type material: Holotype Te Papa Tongarewa AI.071920 (MPN-Ony533). Croydon Bush, Southland, New Zealand (−46.057526, 168.845375), 3/XI/2021. R. B. Morris. Paratype Otago Museum IV.159493 (MPN-Ony551).

Remarks: The full geographic range of this taxon is not known, but accumulating observations show that it extends further inland in Southland and Otago.

***Peripatoides otepoti* sp. nov.**

Type locality: Nichols Creek, Dunedin, Otago, New Zealand.

**Figure 18:**
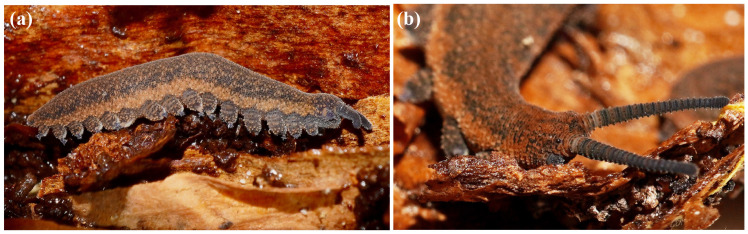
*Peripatoides otepoti* sp. nov. individuals from Nichols Creek, Dunedin. (**a**) Resting position under bark; (**b**) active.

Description: Ovoviviparous ngāokeoke with 15 pairs of legs bearing three distal papillae and three spinous pads. The third spinous pad on the 4th and 5th legs is completely divided by the nephridial tubercle. Adult resting length up to about 30 mm. Colour is dark grey/orange–brown comprising a mixture of pigmented papillae that generally do not form a strong pattern. Individuals often display a predominantly dark grey dorsal midline (Figure 18). The antennae and legs are primarily dark grey. The lower flank is typically with a predominantly orange band with an abrupt dark grey boundary running between the legs. This is most apparent when the specimen is walking.

Most closely related to *P. aurorbis*, *P. taitonga* sp. nov., *P. waikaia* sp. nov. but differs from nearest (*P. taitonga* sp. nov) by 7.3% at the mitochondrial COI gene. Intraspecific diversity in the present data set is 2.2%.

Material examined: MPN-Ony255, Caversham Valley (−45.89459, 170.46795); MPN-Ony260, Dunedin Botanical Gardens (−45.86035, 170.52278); MPN-Ony397, MPN-Ony258, Frasers Gully (−45.86194, 170.46056); MPN-Ony256, Grahams Bush (−45.81777, 170.57187); MPN-Ony268, Gunns Bush (−44.66635, 170.95378); MPN-Ony270, Herbert Forest (−45.24273, 170.77427); MPN-Ony269, Kakanui Mountains (−45.22194, 170.49528); MPN-Ony264, Maungatua (−45.89361, 170.13333); MPN-Ony577, MPN-Ony578, Nichols Creek (−45.83179, 170.49895); MPN-Ony139, Outram (−45.85095, 170.23968); MPN-Ony267, MPN-Ony564, MPN-Ony565, Peel Forest (−43.89528, 171.24833); MPN-Ony265, MPN-Ony266, Saddle Hill (−45.90083, 170.37917); MPN-Ony262, MPN-Ony263, Silver Stream (−45.85095, 170.23968); MPN-Ony259, Styles Creek (−45.84361, 170.66306); MPN-Ony579–581, MPN-Ony254, Tomahawk Lagoon (−45.90027, 170.53909); MPN-Ony261, Trotters Gorge (−45.40444, 170.78222); MPN-Ony257, Whare Flat, (−45.83806, 170.45528). 

Distribution: From southern Canterbury (Peel Forest near the Tara Haoa Range) to the southern limit of Dunedin city in the vicinity of the Taieri River, extending from the coast at Taeiri Mouth inland to Maungatua. It has been observed in native forest, scrub, pine forest, swamp edges and under rocks in the Kakanui Mountains.

Etymology: Ōtepoti is the te reo (Maori language) name given to the location around which Dunedin city was later developed by European settlers. Although not restricted to this location, ngāokeoke from this region have been consistently referenced to Dunedin [2,26,47,48].

Type material: Holotype Te Papa Tongarewa AI.071921 (MPN-Ony578). Nichols Creek, Leith Valley, Dunedin, New Zealand (−45.831973, 170.498292), 24/XII/2022. S. A. Trewick. Paratype Otago Museum IV.159494 (MPN-Ony577).

Remarks: This entity includes the population sometimes referred to as the ‘Caversham peripatus’ [47]. The full geographic range of this taxon is not known, but accumulating observations suggest that it probably extends further inland in Otago and Southern Canterbury. No authenticated records of Onychophora are known from east central Canterbury, Christchurch or Banks Peninsula.

***Peripatoides waikaia* sp. nov.**

Type locality: Piano Flat, Waikaia River, Southland, New Zealand.

**Figure 19:**
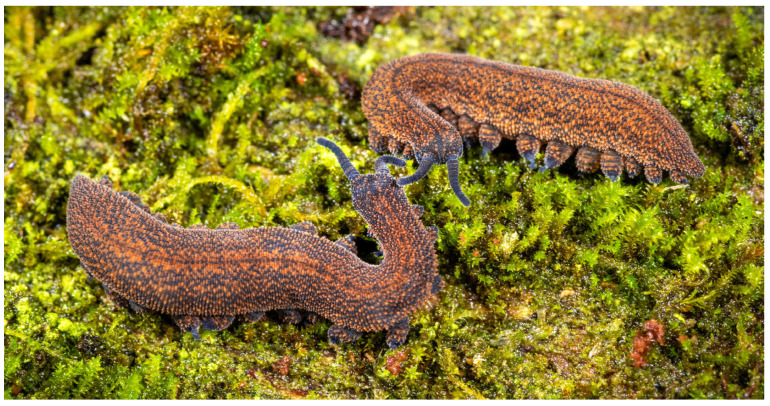
Two *Peripatoides waikaia* sp. nov. from the type locality. © Rod Morris.

Description: Ovoviviparous ngāokeoke with 15 pairs of legs bearing three distal papillae and three spinous pads. The third spinous pad on the 4th and 5th legs is completely divided by the nephridial tubercle. Adult resting length up to about 30 mm. Colour is dark grey/orange–brown comprising an evenly scattered array of pigmented papillae that generally do not form a strong pattern. The head and antennae are predominantly dark grey (Figure 19).

Most closely related to *P. aurorbis*, *P. otepoti* sp. nov., *P. taitonga* sp. nov., but differs from nearest (*P. otepoti* sp. nov) by 8% at the mitochondrial COI gene. Intraspecific diversity in the present data set is 0.1%.

Material examined: MPN-Ony456–462 Piano Flat, Southland (−45.506423, 169.08754); MPN-Ony323, MPN-Ony324 (−45.55557, 169.01938).

Distribution: Currently documented only from native forest towards the head of the Waikaia River.

Etymology: Waikaia is the te reo (indigenous Maori language) name for the river around the remnant native forest where this species has been found.

Type material: Holotype Te Papa Tongarewa AI.071922 (MPN-Ony458). Piano Flat, Waikaia, Southland, New Zealand (−45.553876, 169.031628), 15/VII/2020. R. B. Morris. Paratype Otago Museum IV159495 (MPN-Ony459).

## 4. Discussion

Much of the biodiversity of New Zealand awaits discovery, and the present work contributes to this endeavour by advancing our understanding of ngāokeoke Aotearoa diversity and distribution [47]. Morphological conservatism makes it difficult to differentiate populations of individuals that are biologically distinct in other respects [49]. Where suitable heritable variable characters such as genetic markers are identified, the sympatry of morphologically cryptic populations allows identification of the level of gene flow operating. Wallace [50] pointed out that in the vast majority of cases, it is not possible to confirm reproductive isolation between similar related species as most do not co-occur; however, North Island *Peripatoides* are amenable. By applying a naïve analysis to data for functional, expressed, genetic variation, we found instances where putative population samples actually contained individuals with closer relatives elsewhere, i.e., two or more reproductively isolated species co-occurring in space. 

Species delimitation tools applied to mtDNA sequence data implement the phylogenetic species concept by identifying minimal phylogenetic units [46] with the assumption that gene trees evolve within the constraints of the species tree (no gene flow between incipient species), which is a potential problem for many taxa [51]. We agree with Hillis (2019) [52] that the discovery and description of biodiversity (systematics) can be tackled in two stages: first, forming a hypothesis of how specimens and populations can be grouped together (putative species), and then testing these groupings based on evidence of reproductive isolation [52]. However, in the case of cryptic ngāokeoke, expressed nuclear loci provided direct evidence of reproductive isolation in sympatry first and subsequently, the mtDNA sequence that conveniently identifies cluster membership was found to support the same boundaries [25,26]. Where we do not yet have data from nuclear loci, we rely on the degree of concordance between markers and distinctive colour patterns to provide confidence in forming the species hypotheses [53]. Further work could test these boundaries and resolve the interaction among geographically overlapping lineages. The members of the northern *P. sympatrica*, *P. kawekaenis*, *P. morgani* and *P. novaezealandiae* are closely related and display degrees of overlap on the allopatry to sympatry continuum, which is an expected feature of evolution [54]. A combination of sympatric populations of the most distinct lineages and parapatric populations of less differentiated lineages provides the opportunity to examine the reproductive permeability of species barriers [55,56].

Cryptic diversity within extant Onychophora is not unique to New Zealand, and molecular studies of the onychophorans endemic to Australia, south and central America, and South Africa continue to reveal hidden biodiversity (e.g., [57,58,59,60]). Some Australian taxa display secondary reproductive traits that aid recognition [61,62,63], but in many cases, low character variation is the norm. Among many South African velvet worms, genetic diversity is often not accompanied by diagnostic morphological differentiation [64,65]. Whereas in New Zealand, we detected little (or no) variation within species for numbers of legs, in the South African species *Peripatopsis capensis*, three mtDNA lineages (6% divergence) showed variation in leg number ranging from 17 to 19 pairs [64,66]. Microscopic differentiation can be valuable for testing species hypotheses based on genetic markers (e.g., [67]) and further work with SEM studies might identify diagnostic traits for the new taxa proposed here. Nevertheless, micromorphology is no more compelling in terms of speciation than genetic evidence and shares the limitation of not being detectable for specimens in hand; thus, crypsis remains a problem for biologists. The new ngāokeoke species identified in southern New Zealand are currently known in allopatric populations, but *P. taitonga* sp. nov and *P. otepote* sp. nov. meet in the vicinity of the Taieri River. Landscape features such as rivers may correlate with putative species boundaries, and a role in limiting dispersal has been inferred for velvet worms in South Africa, where the Mkomazi River separates *Peripatopsis birgeri* from *P. polychrome* [67]. Convincingly, establishing the importance of landscape in speciation requires dense sampling of individuals and independent markers [17,21,68].

Sympatry of ngāokeoke most probably results from range shifting, causing species to overlap. Although widespread in North Island, *P. aurorbis* appears to be a southern lineage as intraspecific diversity is highest in the northern South Island. This is consistent with a general pattern of high invertebrate endemicity in this region [69]. The finding of relatively high mtDNA diversity here is as expected if the region has retained a large population size over a period of time or has acted as a refugial nexus [70]. In another New Zealand invertebrate (*Phaulacridium* grasshoppers), it has been found that mtDNA diversity in two species is contrary to their current spatial range sizes due to recent range changes in both species [71]. *Peripatoides aurorbis* may have responded to the opportunity for range expansion during the Pleistocene when lowered sea level (e.g., during the last glacial maximum ~20 kya) resulted in land bridging of Cook Strait [45]. It appears to represent in North Island a southern lineage that is most easily explained by expansion from South Island, and emphasises the dynamism of range change. Contrasting with this is the apparent absence of *Peripatoides* in the central swathe of South Island that was subject to the most intense Pleistocene environmental instability [72]. 

While range shifts and regional extirpation can readily explain complex geographic distributions of species, sympatry creates circumstances where the reproductive compatibility of lineages can be revealed [73]. However, little is yet known about barriers to reproduction in Onychophora. In some taxa, limits to gene flow might result from physical, pheromonal, or behavioural features associated with mating [74], but genomic features are likely to be influential in many others, and this adds to the cryptic nature of these animals. The karyology of several groups of Australian Onychophora suggests chromosome morphology and number are important in lineage formation and/or maintenance [63,75,76,77]. These studies and others that focus on functional genomics have been pivotal in revealing diversity and need further attention to inform our understanding of the evolution and natural history of Onychophora [24,78,79].

Unlike the situation in arid regions [21,80], we find that Onychophora in wetter, temperate environments are successful at moving across the landscape. Despite their apparent frailty, extant Onychophora are being revealed as biodiverse and persistent, with a greater capacity to utilise habitat patches and tolerate natural and anthropogenic land modification than has been widely supposed [80,81,82,83,84].

## Figures and Tables

**Table 1:** Variation of thirteen morphological traits among *Peripatoides* species from central and northern New Zealand.

Species	*n*	Leg Pairs	Orange Papillae $\bar{x}$, Range	Orange Antennal Base	Genital Opening	Spinous Pads	Distal Papillae	Nephridial Tubercle Division		Antenna Rings Range	Papillary Scales Mode, Range	Average Length mm	Colour Pattern		
								4th	5th				Lateral	Ventral	Dorsal
*P. sympatrica*	16	15	23 12–47	Absent, partial, or complete	Blue–grey or orange mix	3	3	Complete or rarely partial	Complete	24–33	8, 6–10	20.7	Absent or partial	Absent, partial or strong	Absent or partial
*P. indigo*	10	15	0	Absent	Blue–grey, rarely orange	4	5	Complete or rarely partial	Complete or rarely partial	30–40	9, 6–12	26.6	None	None	None
*P. aurorbis*	13	15	14.3 4–25	Absent	Orange or very pale	3	3	Complete or rarely partial	Complete	24–33	6, 5–8	17.8	None	Partial or strong	Strong or partial
*P. kawekaensis*	4	15	30.5 24–44	Partial	Blue–grey or orange mix	3	3	Complete or rarely partial	Complete or rarely partial	20–31	6, 6–10	19.3	Absent or partial	Partial or strong	Absent or partial
*P. suteri*	3	16	15.7 15–17	Absent	Blue–grey	3	4	Partial, rarely complete	Complete	27–32	6, 6–10	33	Absent	Absent	Absent
*P. novaezealandiae*	8	15	20.4 15–34	Absent or partial	Blue–grey	3	3	Partial, rarely complete	Partial, rarely complete	21–35	8, 7–10	24.4	Absent, partial or strong	Absent, partial or strong	Absent, partial or strong
*P. morgani*	8	15	22.1 12–38	Partial or absent	Blue–grey or orange mix	3	3	Partial or complete	Partial or complete	24–32	8, 6–10	24.4	Absent, partial or strong	Absent, partial or strong	Absent or partial

**Table 2:** Mitochondrial COI haplotype diversity in New Zealand *Peripatoides*, including the number of haplotypes (h), haplotype diversity (Hd), nucleotide diversity (Pi), and mean intraspecific divergence (HKY).

Species	n	h	Hd	Pi	HKY
*P. sympatrica*	129	69	0.984	0.01613	0.0194
*P. indigo*	4	3	0.833	0.00191	0.0019
*P. aurorbis*	43	33	0.986	0.0322	0.0333
*P. kawekaensis*	26	16	0.938	0.01287	0.0130
*P. suteri*	18	12	0.948	0.01752	0.0189
*P. novaezealandiae*	24	18	0.975	0.01989	0.0204
*P. morgani*	29	16	0.938	0.02075	0.0212
*P. taitonga* sp. nov.	10	10	1.0	0.0197	0.0210
*P. otepote* sp. nov.	20	13	0.926	0.02087	0.0214
*P. waikaia* sp. nov.	6	2	0.333	0.00127	0.0013

## Data Availability

DNA sequence data are available via NCBI-GenBank.

## Supplementary Materials

The following supporting information can be downloaded at: https://www.mdpi.com/article/10.3390/insects15040248/s1, Table S1: Sample details.
