# Supplementary material for: Ngāokeoke Aotearoa: The Peripatoides Onychophora of New Zealand
Source: Insects. 2024 Apr 4;15(4):248. doi: 10.3390/insects15040248 (PMC11050097; doi:10.3390/insects15040248)
Supplement: Supplementary file 1 [file insects-15-00248-s001.zip › insects-2923273-supplementary.pdf]

Table S1: Sample details.

Sample code	Old code	species	latitude	longitude
MPN-Ony022		*suteri*	-39.07161	174.08378
MPN-Ony027		*suteri*	-36.9069	174.5644
MPN-Ony033	DF 1.1	*suteri*	-39.32528	174.1075
MPN-Ony063	RO 1.2	*suteri*	-39.45194	174.40806
MPN-Ony272	CP28	*suteri*	-36.98806	175.62028
MPN-Ony344		*suteri*	-39.79639	174.92782
MPN-Ony391		*suteri*	-39.79639	174.92782
MPN-Ony484		*suteri*	-39.45407	174.41199
MPN-Ony485		*suteri*	-39.45407	174.41199
MPN-Ony489		*suteri*	-39.15359	174.71406
MPN-Ony491		*suteri*	-39.15359	174.71406
MPN-Ony493		*suteri*	-39.15359	174.71406
MPN-Ony539		*suteri*	-39.3598	174.1396
MPN-Ony540		*suteri*	-39.3598	174.1396
MPN-Ony541		*suteri*	-39.32509	174.10578
MPN-Ony542		*suteri*	-39.32509	174.10578
MPN-Ony543		*suteri*	-39.32509	174.10578
MPN-Ony544		*suteri*	-39.32509	174.10578
MPN-Ony348		*indigo*	-40.90176	172.43303
MPN-Ony351		*indigo*	-40.90176	172.43303
MPN-Ony352		*indigo*	-40.90176	172.43303
MPN-Ony353		*indigo*	-40.90176	172.43303
MPN-Ony390		*indigo*	-40.90176	172.43303
MPN-Ony 001	KI 11.1	*aurorbis*	-36.43805	174.85361
MPN-Ony 004	KI 12.1	*aurorbis*	-36.43805	174.85361
MPN-Ony 005	KI 12.2	*aurorbis*	-36.43805	174.85361
MPN-Ony 006	KI 12.3	*aurorbis*	-36.43805	174.85361
MPN-Ony 026		*aurorbis*	-39.23866	175.81221
MPN-Ony 065	PU 2.2	*aurorbis*	-38.56667	175.71667
MPN-Ony 147		*aurorbis*	-37.02639	175.5355
MPN-Ony228		*aurorbis*	-41.29806	173.57166
MPN-Ony229		*aurorbis*	-41.45861	173.70417
MPN-Ony230		*aurorbis*	-41.45861	173.70417
MPN-Ony246	KI13.1	*aurorbis*	-36.43805	174.85361
MPN-Ony247	RT3.1	*aurorbis*	-38.06107	176.71543
MPN-Ony248	CP29	*aurorbis*	-36.98806	175.62028
MPN-Ony249	COB1	*aurorbis*	-41.13333	172.62806
MPN-Ony320	FS2.1	*aurorbis*	-41.19001	172.741
MPN-Ony321	PB1.1	*aurorbis*	-41.29806	173.57166
MPN-Ony326	PYR1	*aurorbis*	-41.17961	172.95985
MPN-Ony327	WK1.1	*aurorbis*	-36.90194	174.53806
MPN-Ony336		*aurorbis*	-40.83615	172.45231

MPN-Ony339		*aurorbis*	-41.31319	173.02577
MPN-Ony340		*aurorbis*	-41.28436	172.70095
MPN-Ony383		*aurorbis*	-38.15413	175.69465
MPN-Ony384		*aurorbis*	-38.15413	175.69465
MPN-Ony387		*aurorbis*	-40.86726	173.03407
MPN-Ony410		*aurorbis*	-41.45861	173.70417
MPN-Ony413		*aurorbis*	-41.45861	173.70417
MPN-Ony467		*aurorbis*	-41.19013	172.74576
MPN-Ony468		*aurorbis*	-41.19013	172.74576
MPN-Ony490		*aurorbis*	-39.15359	174.71406
MPN-Ony492		*aurorbis*	-39.15359	174.71406
MPN-Ony495		*aurorbis*	-38.88338	175.30814
MPN-Ony497		*aurorbis*	-38.88338	175.30814
MPN-Ony499		*aurorbis*	-38.88338	175.30814
MPN-Ony501		*aurorbis*	-38.88338	175.30814
MPN-Ony503		*aurorbis*	-38.88338	175.30814
MPN-Ony521		*aurorbis*	-38.95115	175.18824
MPN-Ony522		*aurorbis*	-38.95115	175.18824
MPN-Ony523		*aurorbis*	-38.95115	175.18824
MPN-Ony527		*aurorbis*	-38.64672	175.66023
MPN-Ony528		*aurorbis*	-38.64672	175.66023
MPN-Ony536		*aurorbis*	-39.64756	175.72316
MPN-Ony254	TOM1	*otepote* sp. nov.	-45.90027	170.53909
MPN-Ony255	CV1.2	*otepote* sp. nov.	-45.89459	170.46795
MPN-Ony256	GB1.1	*otepote* sp. nov.	-45.81777	170.57187
MPN-Ony257	Whf1.1	*otepote* sp. nov.	-45.83806	170.45528
MPN-Ony258	FG1.3	*otepote* sp. nov.	-45.86194	170.46056
MPN-Ony259	SCB1.1	*otepote* sp. nov.	-45.84361	170.66306
MPN-Ony260	DBG1.3	*otepote* sp. nov.	-45.86	170.52278
MPN-Ony261	TG1	*otepote* sp. nov.	-45.40444	170.78222
MPN-Ony262	SS1.1	*otepote* sp. nov.	-45.85095	170.23968
MPN-Ony263	SS1.2	*otepote* sp. nov.	-45.85095	170.23968
MPN-Ony264	MAU1	*otepote* sp. nov.	-45.89361	170.13333
MPN-Ony265	SH1.2	*otepote* sp. nov.	-45.90083	170.37917
MPN-Ony266	SH1.3	*otepote* sp. nov.	-45.90083	170.37917
MPN-Ony267	PEE6	*otepote* sp. nov.	-43.89528	171.24833
MPN-Ony268	GU3	*otepote* sp. nov.	-44.66583	170.965
MPN-Ony269	KAK1	*otepote* sp. nov.	-45.22194	170.49528
MPN-Ony270	Herb1	*otepote* sp. nov.	-45.24273	170.77427
MPN-Ony397		*otepote* sp. nov.	-45.86194	170.46056
MPN-Ony564		*otepote* sp. nov.	-43.89528	171.24833
MPN-Ony565		*otepote* sp. nov.	-43.89528	171.24833
MPN-Ony566		*otepote* sp. nov.	-43.89528	171.24833
MPN-Ony577		*otepote* sp. nov.	45.83211	170.49889
MPN-Ony578		*otepote* sp. nov.	45.83211	170.49889

MPN-Ony579		*otepote* sp. nov.	-45.90027	170.53909
MPN-Ony580		*otepote* sp. nov.	-45.90027	170.53909
MPN-Ony581		*otepote* sp. nov.	-45.90027	170.53909
MPN-Ony029		*morgani*	-39.965	176.28194
MPN-Ony035	MK 3.1	*morgani*	-39.965	176.28194
MPN-Ony036	RA 3.1	*morgani*	-39.0005	176.54316
MPN-Ony038	MO 5.1	*morgani*	-39.8575	176.88823
MPN-Ony039	NW 4.3	*morgani*	-40.50611	176.2225
MPN-Ony054	OU 1.2	*morgani*	-40.07831	176.67396
MPN-Ony082	MK 3.2	*morgani*	-39.96363	176.28147
MPN-Ony083	NW 4.2	*morgani*	-40.50611	176.2225
MPN-Ony152		*morgani*	-40.50611	176.2225
MPN-Ony223		*morgani*	-39.91917	176.8177
MPN-Ony224		*morgani*	-39.91917	176.8177
MPN-Ony275	MO1.1	*morgani*	-39.85738	176.90306
MPN-Ony276	CA1.1	*morgani*	-41.00167	175.59833
MPN-Ony277	CA2.3	*morgani*	-41.00167	175.59833
MPN-Ony301		*morgani*	-39.85738	176.90306
MPN-Ony304		*morgani*	-39.85738	176.90306
MPN-Ony308		*morgani*	-39.85738	176.90306
MPN-Ony329		*morgani*	-39.65542	177.03724
MPN-Ony017		*novaezealandiae*	-40.42722	176.12944
MPN-Ony019		*novaezealandiae*	-41.30806	174.89861
MPN-Ony034	MI 1.1	*novaezealandiae*	-40.70639	175.65277
MPN-Ony052	OT 3.2	*novaezealandiae*	-41.2655	174.75583
MPN-Ony129		*novaezealandiae*	-40.81753	175.16327
MPN-Ony 130		*novaezealandiae*	-40.81753	175.16327
MPN-Ony 131		*novaezealandiae*	-40.7988	175.19639
MPN-Ony 135		*novaezealandiae*	-41.2655	174.75583
MPN-Ony 141		*novaezealandiae*	-40.638826	175.32369
MPN-Ony 142		*novaezealandiae*	-40.638826	175.32369
MPN-Ony 143		*novaezealandiae*	-40.638826	175.32369
MPN-Ony212		*novaezealandiae*	-40.87247	175.2338
MPN-Ony213		*novaezealandiae*	-40.87247	175.2338
MPN-Ony215		*novaezealandiae*	-40.87247	175.2338
MPN-Ony232		*novaezealandiae*	-41.27722	174.9733
MPN-Ony274	PA3.2	*novaezealandiae*	-40.46194	175.78611
MPN-Ony357		*novaezealandiae*	-40.83389	174.93373
MPN-Ony439		*novaezealandiae*	-40.648029	175.23326
MPN-Ony573		*novaezealandiae*	-40.96996	174.97355
MPN-Ony025		*kawekaensis*	-39.9649	175.52432
MPN-Ony045	RA 2.1	*kawekaensis*	-38.93111	176.49028
MPN-Ony074	HU 4.1	*kawekaensis*	-39.27166	176.54139
MPN-Ony133		*kawekaensis*	-39.9649	175.52432
MPN-Ony285	NW2.1	*kawekaensis*	-40.50611	176.2225

MPN-Ony286	TA4	*kawekaensis*	-39.30694	176.88361
MPN-Ony297		*kawekaensis*	-39.11944	176.8088
MPN-Ony309		*kawekaensis*	-39.11166	176.81806
MPN-Ony331		*kawekaensis*	-39.97444	176.00667
MPN-Ony338		*kawekaensis*	-38.86247	176.88714
MPN-Ony419		*kawekaensis*	-39.1266	176.80676
MPN-Ony513		*kawekaensis*	-39.90286	175.60099
MPN-Ony514		*kawekaensis*	-39.90286	175.60099
MPN-Ony515		*kawekaensis*	-39.90286	175.60099
MPN-Ony533		*kawekaensis*	-39.64756	175.72316
MPN-Ony535		*kawekaensis*	-39.64756	175.72316
MPN-Ony538		*kawekaensis*	-39.64756	175.72316
MPN-Ony575	BC3.1	*kawekaensis*	-39.27	176.49833
MPN-Ony250	TM1.1	*taitonga* sp. nov.	-46.05256	170.19057
MPN-Ony251	CAT1.1	*taitonga* sp. nov.	-46.60722	169.01556
MPN-Ony252	DA1	*taitonga* sp. nov.	-46.05762	168.82578
MPN-Ony253	TC4.1	*taitonga* sp. nov.	-45.85123	169.4715
MPN-Ony325	MF1.1	*taitonga* sp. nov.	-46.49821	169.49747
MPN-Ony463		*taitonga* sp. nov.	-45.91539	169.99274
MPN-Ony551		*taitonga* sp. nov.	-46.0633	168.87371
MPN-Ony552		*taitonga* sp. nov.	-46.0633	168.87371
MPN-Ony553		*taitonga* sp. nov.	-46.0633	168.87371
MPN-Ony555		*taitonga* sp. nov.	-46.36253	168.70472
MPN-Ony556		*taitonga* sp. nov.	-46.36253	168.70472
MPN-Ony557		*taitonga* sp. nov.	-46.36253	168.70472
MPN-Ony558		*taitonga* sp. nov.	-46.36253	168.70472
MPN-Ony560		*taitonga* sp. nov.	-45.89388	169.35048
MPN-Ony561		*taitonga* sp. nov.	-45.89388	169.35048
MPN-Ony562		*taitonga* sp. nov.	-45.89388	169.35048
MPN-Ony576	TM3	*taitonga* sp. nov.	-46.05256	170.19057
MPN-Ony323	PF1.1	*waikaia* sp. nov.	-45.55557	169.01938
MPN-Ony324	PF1.2	*waikaia* sp. nov.	-45.55557	169.01938
MPN-Ony457		*waikaia* sp. nov.	-45.506423	169.08754
MPN-Ony458		*waikaia* sp. nov.	-45.506423	169.08754
MPN-Ony459		*waikaia* sp. nov.	-45.506423	169.08754
MPN-Ony460		*waikaia* sp. nov.	-45.506423	169.08754
MPN-Ony461		*waikaia* sp. nov.	-45.506423	169.08754
MPN-Ony018		*sympatrica*	-40.42722	176.12944
MPN-Ony023		*sympatrica*	-39.43055	175.5211
MPN-Ony024		*sympatrica*	-39.01722	175.73361
MPN-Ony040	NW 4.1	*sympatrica*	-40.50611	176.2225
MPN-Ony057	SR 3.2	*sympatrica*	-40.29388	175.8025
MPN-Ony060	FB 2.1	*sympatrica*	-37.14917	175.70083
MPN-Ony071	TH 1.1	*sympatrica*	-37.1388	175.60694
MPN-Ony123		*sympatrica*	-38.76472	176.2144

MPN-Ony127		*sympatrica*	-38.76472	176.2144
MPN-Ony132		*sympatrica*	-39.9649	175.52432
MPN-Ony137		*sympatrica*	-39.35985	175.47237
MPN-Ony138		*sympatrica*	-39.35985	175.47237
MPN-Ony148		*sympatrica*	-38.55607	175.73622
MPN-Ony149		*sympatrica*	-39.20361	175.53861
MPN-Ony150		*sympatrica*	-38.74917	177.16139
MPN-Ony153		*sympatrica*	-40.50611	176.2225
MPN-Ony156		*sympatrica*	-38.74917	177.16139
MPN-Ony160		*sympatrica*	-37.67934	177.76574
MPN-Ony161		*sympatrica*	-38.04451	178.31626
MPN-Ony163		*sympatrica*	-38.59583	177.95917
MPN-Ony165		*sympatrica*	-37.5975	178.29917
MPN-Ony167		*sympatrica*	-37.5975	178.29917
MPN-Ony169		*sympatrica*	-37.5975	178.29917
MPN-Ony171		*sympatrica*	-37.5975	178.29917
MPN-Ony172		*sympatrica*	-40.50083	175.5088
MPN-Ony177		*sympatrica*	-37.5388	175.73528
MPN-Ony178		*sympatrica*	-37.5388	175.73528
MPN-Ony183		*sympatrica*	-40.91472	175.66306
MPN-Ony185		*sympatrica*	-40.91472	175.66306
MPN-Ony191		*sympatrica*	-38.40917	177.4066
MPN-Ony195		*sympatrica*	-38.40917	177.4066
MPN-Ony197		*sympatrica*	-38.40917	177.4066
MPN-Ony217		*sympatrica*	-39.8575	176.88823
MPN-Ony218		*sympatrica*	-39.8575	176.88823
MPN-Ony219		*sympatrica*	-39.91917	176.8177
MPN-Ony220		*sympatrica*	-39.91917	176.8177
MPN-Ony221		*sympatrica*	-39.91917	176.8177
MPN-Ony222		*sympatrica*	-39.91917	176.8177
MPN-Ony231		*sympatrica*	-38.76472	176.2144
MPN-Ony233		*sympatrica*	-36.50917	175.42139
MPN-Ony234		*sympatrica*	-36.50917	175.42139
MPN-Ony243		*sympatrica*	-40.3155	175.78055
MPN-Ony244		*sympatrica*	-40.3155	175.78055
MPN-Ony287	NG2.1	*sympatrica*	-35.30111	174.25306
MPN-Ony289	NW1.1	*sympatrica*	-40.50611	176.2225
MPN-Ony290	WO1	*sympatrica*	-38.26517	175.07964
MPN-Ony291	RK3	*sympatrica*	-37.92278	177.92083
MPN-Ony292	NG4.1	*sympatrica*	-35.30111	174.25306
MPN-Ony293	HO1.1	*sympatrica*	-35.26689	173.20843
MPN-Ony294	WP1.2	*sympatrica*	-35.63444	173.55806
MPN-Ony295	WP1.1	*sympatrica*	-35.63444	173.55806
MPN-Ony296	PK1.1	*sympatrica*	-35.27694	173.6825
MPN-Ony298		*sympatrica*	-38.74917	177.16139

MPN-Ony300	*sympatrica*	-38.26517	175.07964
MPN-Ony302	*sympatrica*	-39.85738	176.90306
MPN-Ony303	*sympatrica*	-39.85738	176.90306
MPN-Ony305	*sympatrica*	-39.85738	176.90306
MPN-Ony306	*sympatrica*	-39.85738	176.90306
MPN-Ony311	*sympatrica*	-38.72459	177.07456
MPN-Ony314	*sympatrica*	-36.63547	175.80785
MPN-Ony315	*sympatrica*	-36.63547	175.80785
MPN-Ony317	*sympatrica*	-39.79744	176.19641
MPN-Ony330	*sympatrica*	-39.98054	176.00687
MPN-Ony382	*sympatrica*	-38.15413	175.69465
MPN-Ony385	*sympatrica*	-38.15413	175.69465
MPN-Ony386	*sympatrica*	-38.15413	175.69465
MPN-Ony392	*sympatrica*	-39.79748	176.19667
MPN-Ony400	*sympatrica*	-35.19823	173.7983
MPN-Ony401	*sympatrica*	-35.19823	173.7983
MPN-Ony402	*sympatrica*	-35.19823	173.7983
MPN-Ony403	*sympatrica*	-35.19823	173.7983
MPN-Ony428	*sympatrica*	-40.91472	175.66306
MPN-Ony429	*sympatrica*	-40.91472	175.66306
MPN-Ony445	*sympatrica*	-35.70247	174.26436
MPN-Ony446	*sympatrica*	-35.70247	174.26436
MPN-Ony448	*sympatrica*	-35.70247	174.26436
MPN-Ony449	*sympatrica*	-35.70247	174.26436
MPN-Ony451	*sympatrica*	-35.19293	173.48057
MPN-Ony452	*sympatrica*	-35.19293	173.48057
MPN-Ony453	*sympatrica*	-35.19293	173.48057
MPN-Ony505	*sympatrica*	-38.86144	175.5352
MPN-Ony508	*sympatrica*	-38.86144	175.5352
MPN-Ony509	*sympatrica*	-38.86144	175.5352
MPN-Ony510	*sympatrica*	-38.86144	175.5352
MPN-Ony518	*sympatrica*	-39.92808	175.19614
MPN-Ony519	*sympatrica*	-39.92808	175.19614
MPN-Ony520	*sympatrica*	-39.92808	175.19614
MPN-Ony525	*sympatrica*	-38.64672	175.66023
MPN-Ony526	*sympatrica*	-38.64672	175.66023
MPN-Ony530	*sympatrica*	-38.64672	175.66023
MPN-Ony531	*sympatrica*	-38.64672	175.66023
